# Hybrid-silica nanoparticles as a delivery system of the natural biocide carvacrol

**DOI:** 10.1039/c8ra05898a

**Published:** 2018-10-30

**Authors:** Chana G. Sokolik, Jean-Paul Lellouche

**Affiliations:** Department of Chemistry, Institute of Nanotechnology & Advanced Materials (BINA), Bar-Ilan University Ramat-Gan 5290002 Israel lellouj@biu.ac.il

## Abstract

Bacterial resistance to common antibiotics necessitates innovative solutions. The phenolic antimicrobial compound carvacrol, a major ingredient in the Essential Oils (EOs) of oregano and thyme, has the advantages of natural compounds such as Generally Recognized As Safe (GRAS) status, but needs an appropriate delivery system designed to overcome its drawbacks (such as low aqueous solubility, easy phenol oxidation, heat/light inactivation, distinct odor). An alkoxysilane incorporating the carvacrol moiety is synthesized and subsequently employed to fabricate hybrid silica nanoparticles (NPs) with carvacrol covalently bound to the silica matrix. The enzymatically hydrolyzable carbamate bond turns these NPs into a release-on-demand nanoscale system for the biocide carvacrol. Characterization of both silane linker and hybrid silica NPs, including quantification of the bioactive compound in the bulk and on the NP surface, is accomplished by spectroscopic methods, including X-ray Photoelectron Spectroscopy (XPS), and Thermo-Gravimetric Analysis (TGA), Dynamic Light Scattering (DLS), *ζ*-potential measurements, as well as electron microscopy. Preliminary biological testing with *E. coli* proves an antibacterial effect. The carbamoylation reaction employed to synthesize the hybrid silica precursor might be readily applied to other bioactive phenolic compounds.

## Abstract 2

The authors fabricated hybrid-silica NPs incorporating the natural antibiotic carvacrol from essential oils covalently with an enzymatically cleavable bond. They are a safe, on-demand antibacterial agent.

## Graphical abstract

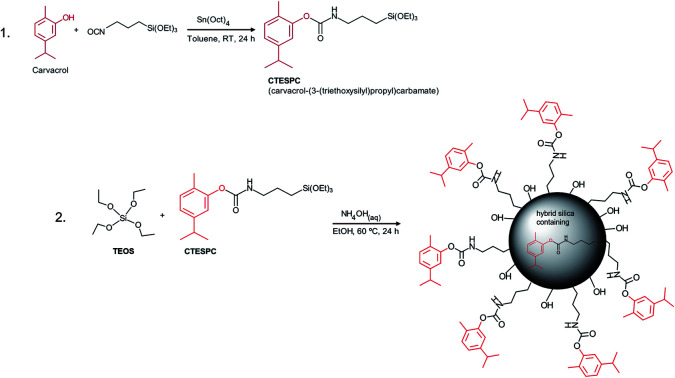

## Introduction

The significance of antibacterial agents is not limited to strictly pharmaceutical applications for treating and preventing infectious diseases. Many consumer products such as food, cosmetics, and cleaning materials also contain antibacterial agents as standard ingredients to prolong shelf life, maintain freshness, control foodborne pathogens, promote hygiene, and more. However, the widespread use of antibiotic agents leads to emergence of resistant strains of bacteria. Today, there are strains of bacteria that are practically resistant to all common antibiotics. The development of new antibiotics is an extremely expensive enterprise with a very lengthy approval process. Therefore, the World Health Organization warns that we may be heading towards an era in which common infections and minor injuries can kill again.^1^

Antibacterial substances are found in nature in many plants, though, presumably as part of their defense mechanism against harmful microorganisms and reactive oxygen species. One class of such substances is Essential Oils (EOs). Fragrant volatile compounds derived from spice plants compose the EOs. Most EO compounds are terpenes and terpenoids.^2^

EOs and individual EO compounds are attractive candidates for enlisting in the campaign against resistance. Because they are readily available from natural sources, their cost is relatively low. They have fewer side effects and lower toxicity than non-natural products and are usually classified as GRAS (Generally Recognized As Safe) by the FDA.^3^ EOs and their compounds have better biodegradability than non-natural antibiotics and preservatives.^4^ The urgency of developing successful EO applications is heightened as concern grows about the safety of using some common broad-spectrum antibiotics, such as triclosan. Triclosan, the use of which was once pervasive in consumer products, was recently banned (among other active ingredients) by the FDA for use in soaps because of potential health risks, including bacterial resistance and hormonal effects.^5^ There are also concerns about its bio-accumulation and toxic byproducts.^6,7^

Because EO compounds affect several cellular targets, they are not likely to induce resistance easily. There also seems to be not much threat of single genetic mutations or series of mutations making the membranes impermeable for EOs because such mutations would probably not allow normal membrane and cell functioning.^8^ Furthermore, several EO compounds may work in synergy with antibiotics through multi-target effects or targeting bacterial resistance mechanisms, for example by inhibiting membranal efflux pumps and protective enzymes. The required dose of antibiotics is then lowered, which can mean that bacteria that had become resistant to a certain antibiotic become again susceptible to the same antibiotic when the EO compound is also present.^9–11^

The mechanism of the antimicrobial effect of EOs is based on their lipophilicity, which enables them to interact with cell membranes. The EOs place themselves between the lipid chains, and, as a result, the membranes become more fluid.^12,13^ This increases the membrane's permeability and disrupts cell homeostasis. As a result, the pH in the cell and the membrane potential are reduced, which impacts many cell processes, such as ATP formation.^2,12^ Specifically for carvacrol, active ion transport through the membrane *via* the hydroxyl group has also been proposed.^13^ In addition, research suggests that EOs, among them carvacrol, can interfere with biofilm formation^14,15^ and inhibit growth of existing biofilms.^14^

Besides antibacterial activity, EOs also exhibit other promising qualities. Specifically, they show anti-inflammatory,^4^ antioxidant,^4,16,17^ antiradical,^16^ anti-mutagenic,^2^ anti-angiogenic,^17,18^ anti-carcinogenic,^4,19,20^ antiviral,^21^ antimycotic,^22–25^ antiparasitic,^26,27^ and insecticidal activities.^28,29^

However, alongside their advantages, all EO compounds present significant challenges for antibacterial applications. They easily evaporate and, due to their hydrophobicity, have low solubility in aqueous media. In addition, they are susceptible to degradation through exposure to oxygen, UV light, moisture, and heat. To overcome some or all of these problems, two main approaches have been followed: (a) forming nano- and micro-emulsions of EO compounds or (b) encapsulating EO compounds in (modified) biopolymers,^30–33^ sol–gel materials,^34,35^ or other porous materials such as MOFs (metal–organic frameworks).^36^ In both of these approaches, a system is formed that non-covalently incorporates the EO compound within a matrix. Regarding covalent attachment, eugenol and carvacrol-related aldehydes have been grafted onto chitosan NPs.^37^ Also, eugenol, thymol, and carvacrol have been grafted onto silica microparticles *via* aldehyde derivatization.^38^

Encapsulation based on inorganic silica makes use of the special properties of silica and the advantages of sol–gel synthesis as a fabrication method. Amorphous silica is regarded as nontoxic, biocompatible, and biodegradable.^39^ Therefore, its use is already established in many applications.^40,41^ The mild conditions of sol–gel synthesis preserve the full activity of the encapsulated active compound. On the other hand, the inorganic silica matrix offers protection from degrading factors, such as UV light and oxygen, often making the active compound more effective than the free compound. Sol–gel synthesis is a simple, low-cost, and easily scaled-up process. EO-derived compounds and EOs have been encapsulated within silica pores or within inclusion complexes (for example of β-cyclodextrin) that are in turn encapsulated within a silica phase.^42^ However, because the EO compound is not bound covalently, it may leak out of the pores prematurely and too fast. We, therefore, were interested in developing a silica-based delivery system in which the EO compound is covalently bound and released only when needed, through exposure to bacterial enzymes. This minimal release leads to a sustained antibacterial effect and to significant odor-masking (which is an important advantage in many applications involving EOs when the typically strong odor might be undesirable).

**Fig. 1:**
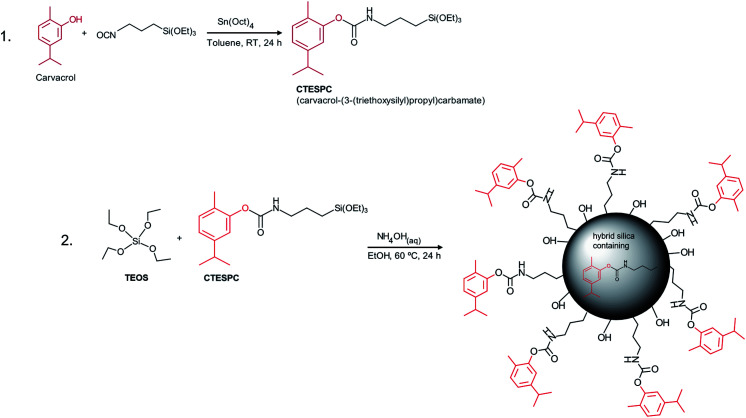
Reaction scheme of (1) CTESPC synthesis and (2) hybrid silica NP fabrication.

For the EO compound in our research, we chose the phenolic carvacrol because of its relatively high antibacterial activity. It is classified by the FDA as Generally Recognized As Safe (GRAS).^3,43^ It can comprise up to 80% of oregano oil and 2–11% of thyme oil, the exact amount depending on various factors.^12^ Concerning synthetic covalent derivatization, its phenolic hydroxyl group enables easy synthesis of an organo-functional alkoxysilane that can undergo co-condensation to form hybrid silica materials (see Fig. 1).

## Experimental

### Chemicals and reagents

Carvacrol (≥98%, FCC, FG), toluene (99.8%, anhydr.), ammonium hydroxide (ACS reagent, 28.0–30.0%), tetraethyl orthosilicate (TEOS) (99.999%) and 3-(triethoxysilyl)propyl isocyanate (95%) were purchased from Sigma-Aldrich, tetraoctyltin (for synthesis) from Merck and ethanol for synthesis from Bio-Lab (abs., AR). All were used without further purification.

### Synthesis of carvacrol-(3-(triethoxysilyl)propyl)carbamate (CTESPC)

Carvacrol (1.078 ml) (7.00 mmol = 1 eq.) was put into an oven-dried 3-necked, round-bottom flask (100 ml) under nitrogen and toluene anhydr. (10 ml) was added (yielding a 0.7 M solution of carvacrol). Stirring was started and 3-(triethoxysilyl)propyl isocyanate (2.600 ml, 10.5 mmol = 1.5 eq.) and tetraoctyltin (CH_3_(CH_2_)_74_Sn, 6.124 ml, 10.5 mmol = 1.5 eq.) were added. Stirring was continued at room temperature under nitrogen atmosphere for at least 15 hours. A thin-layer chromatography (TLC) plate showed the presence of the product (9 : 1 *n*-hexane : ethyl acetate (EtOAc), silica plate, *R*_f_ of product 0.1). After stripping of toluene the product was separated through silica column chromatography (at first 10 : 1 *n*-hexane : EtOAc, after elution of all components of the crude mixture except for the product, 6 : 1 *n*-hexane : EtOAc and then 4 : 1 until elution of CTESPC).

After stripping of the solvent, a clear oil is obtained. Storage in the desiccator turns it into a waxy solid. Yield is 35–54%.

### NMR data of carvacrol-(3-(triethoxysilyl)propyl)carbamate (CTESPC)

^1^H NMR (300 MHz, CDCl_3_, 25 °C, tetramethylsilane (TMS) as reference) *δ* = 7.11 (d, *J* = 7.8 Hz, 1H, (Me)_2_CHCC***H***CH), 6.98 (d, *J* = 7.8 Hz, 1H, (Me)_2_CHCCHC***H***), 6.92 (s, 1H, (Me)_2_CHCC***H***C(O)), 5.35 (bt, 1H, N***H***), 3.84 (q, *J* = 6.9 Hz, 6H, CH_3_C***H***_2_O), 3.28 (m, 2H, NHC***H***_2_), 2.86 (sept, *J* = 6.9 Hz, 1H, (Me)_2_C***H***), 2.17 (s, 3H, CHC(C***H***_3_)), 1.71 (m, 2H, NHCH_2_C***H***_2_), 1.24 (t, *J* = 6.9 Hz, 9H, C***H***_3_CH_2_O), 1.22 (d, *J* = 6.9 Hz, 6H, (C***H***_3_)_2_CH), 0.69 (m, 2H, SiC***H***_2_) ^13^C NMR (150.9 MHz, CDCl_3_, 25 °C, TMS) *δ* = 154.6 (1C, NH***C*** = O(O)), 149.4 (1C, NHC=O(O)***C***), 147.9 (1C, (O)CCH***C***(iPr)), 130.7 (1C, CH***C***(Me)C), 127.7 (1C, (Me)CCH***C***HC(iPr)), 123.6 (1C, (Me)C***C***HCHC(iPr)), 120.2 (1C, (Me)CC(O)***C***H), 58.5 (3C, CH_3_***C***H_2_O), 43.6 (1C, NH***C***H_2_), 33.6 (1C, (Me)_2_***C***H), 23.9 (1C, NHCH_2_***C***H_2_), 23.2 (2C, (***C***H_3_)_2_CH), 18.3 (1C, (***C***H_3_)C), 15.7 (3C, ***C***H_3_CH_2_O), 7.7 (1C, Si***C***H_2_).

### Fabrication of hybrid-silica NPs containing carvacrol by Co-condensation *via* a modified Stöber method

The amount of CTESPC in the reaction mixture is written as % *n*/*n* and is calculated according to:
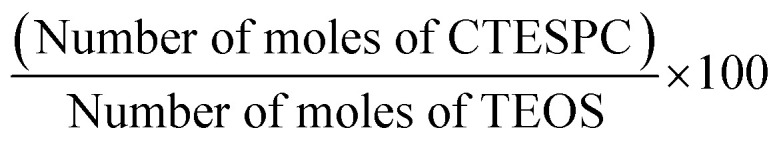


CTESPC was weighed into a 20 ml vial (needed weight calculated according to % *n*/*n* that is desired), and EtOH abs. (10 ml) was added. At high % *n*/*n*, the resulting solution was put into a preheated shaker bath at 50 °C for 10 min to improve solubility. TEOS (0.400 ml) and then NH_4_OH_(aq)_ (28–30%, 0.800 ml) was added with vortex mixing after every addition.

The vial was put on an orbital shaker at RT (250 rpm) or at ≥15% *n*/*n* into a shaker bath at 60 °C (180–220 rpm) for ∼24 hours.

The washing and centrifugation cycles were: 3–4 times H_2_O with ∼10% EtOH to reduce the pH and remove some of the unreacted CTESPC, 3 times EtOH to remove remaining CTESPC and TEOS (13 000–13 500 rpm, 20 min, 3 °C). If DLS size and *ζ*-potential were measured in H_2_O, another time washing in H_2_O followed. For biological testing, the NPs were washed another 3 times with H_2_O. For analyses that require a dry powder and/or for quantification of the NPs (mg dry NPs/ml), the washed NPs were re-dispersed in 1.5–3 ml H_2_O, the dispersion frozen in liquid N_2_ and lyophilized to dryness (0.001 mbar, 24 hours, −106 °C).

### Fabrication of similar non-hybrid silica NPs by Stöber method as reference for analyses and control in biological testing

EtOH abs. (10 ml) was put in a vial of 20 ml. NH_4_OH_(aq)_ (28–30%, 0.8 ml) and TEOS (0.4 ml) were added with vortex mixing after every addition. The mixture was put on an orbital shaker for 24 hours at RT. The resulting NPs were washed 3 times with H_2_O and 3 times with EtOH. The remaining details are identical to those of the hybrid-silica NPs.

### Biological testing

*Escherichia coli* 8739 were grown for 20–24 hours in Nutrient Broth (NB, Sigma) media under shaking (250 rpm) at 37 °C. The following day, the overnight cultures were diluted in fresh NB medium to obtain a stock solution with a concentration of 50 × 10^5^ Colony Forming Units (CFU) per ml (according to the relation of OD_600_ of 1 = 0.8 × 10^9^*E. coli* cells common as standard lab practice in microbiology). The stock solution (20 μl, 10^5^ CFU in 20 μl) was added to the NP solution (1 ml). The resulting solution was incubated at 37 °C for 24 hours. Then serial dilutions were carried out and the cells spotted in duplicate onto NB agar plates. The NB agar plates were incubated at 37 °C for 16–20 hours after which the colonies were counted.

The experimental controls were doubly-deionized water and pure silica NP solutions at approx. the same concentration of mg NPs/ml as the tested solution.

#### Instrumentation

^1^H-NMR spectra were measured on an Avance II Bruker 300 MHz spectrometer at 25 °C in CDCl_3_ with use of Me_4_Si (tetramethylsilane, TMS) as internal standard. ^13^C-NMR spectra were measured on an Avance Bruker DMX 600 MHz spectrometer under the same conditions.

Mass spectroscopy using Electrospray Ionization (ESI) was carried out on an Agilent 6100 Single Quad MS system with methanol as solvent.

An FT-IR spectrometer of Thermo-Scientific with iD7 ATR accessory with a diamond crystal was used for characterization of CTESPC at 100 scans, a resolution of 4 cm^−1^, and a data spacing of 0.482 cm^−1^.

The hydrodynamic particle diameters, size distributions and *ζ*-potential of the particles were measured on a Nano-ZS Zetasizer Nano series of Malvern Instruments, Ltd., UK. For particle size, the measurement conditions were 25 °C (no equilibration time), automatic measurement duration, 3 measurements with up to 12 sub-runs, no delay between measurements and automatic attenuation selection. A disposable cuvette was used. *ζ*-potential measurements were carried out with a minimum of 10 and a maximum of 100 runs at 25 °C. DTS 1070 folded capillary *ζ*-potential cells were filled with sample dispersions (0.8 ml): washed NPs were dispersed in EtOH or H_2_O (1.5–3 ml), diluted 1 : 1.5 with EtOH or H_2_O, and sonicated for 4 min in a low-power sonication bath (Elmasonic S 30 ultrasonic bath, 37 kHz at full power). For DLS measurements, this solution was diluted 1 : 35 with EtOH or H_2_O. For *ζ*-potential measurements, the ratio was 1 : 18 or 1 : 19. In both cases, an additional sonication of 4 min was carried out right before the measurement.

TEM images were obtained on a JEM-1400, JEOL and Tecnai G2, FEI instrument equipped with a Gatan CCD camera, at an operating voltage of 120 kV. For preparation of TEM grids washed NPs were dispersed in EtOH (1.5–3 ml). The resulting suspension was diluted 1 : 1.5 with EtOH and sonicated for 4 min in a low-power sonication bath (Elmasonic S 30 ultrasonic bath, 37 kHz at full power). After an additional dilution 1 : 75 with EtOH and 4 min sonication in the sonication bath, a drop was placed onto a 400 mesh carbon-covered copper grid and dried in air.

HR-SEM images were obtained on a FEI, Magellan 400L. Powder of dried NPs was distributed on double-sided carbon tape pasted onto a copper grid. Then, carbon coating was applied.

**Fig. 2:**
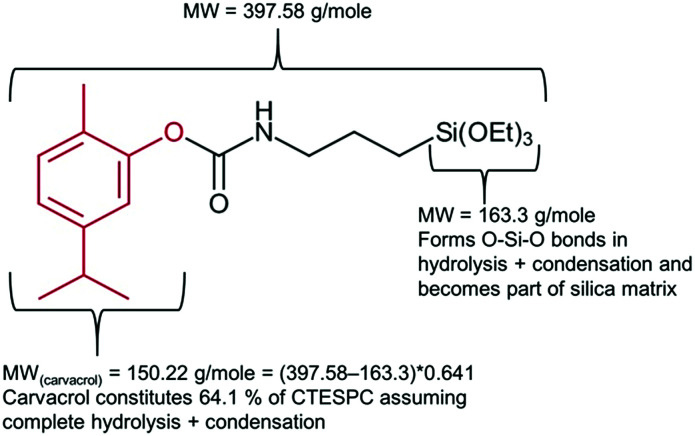
Explanation of factor 0.641 used in calculation of carvacrol content (mg ml^−1^) in CTESPC-containing NPs based on TGA results.

Quantification of carvacrol in the NPs (% w/w) was carried out through measuring the weight loss as a function of temperature. Measurements were carried out on a TGA/DSC 1 STAR^e^ System of Mettler Toledo and the conditions employed were a temperature range of 25–800 °C and a heating rate of 10 °C min^−1^. The gas employed was air at a rate of 50 ml min^−1^. Samples were put into an alumina pan. Since the MW of carvacrol (150.22 g mol^−1^) constitutes 64.1% of the organic part of the CTESPC molecule (see Fig. 2), the weight loss of Step 2 (after the subtraction of the average weight loss of 6 samples of non-hybrid silica NPs in Step 2) is multiplied by 0.641 to obtain an estimate of “mg carvacrol/mg NPs”.

**Fig. 3:**
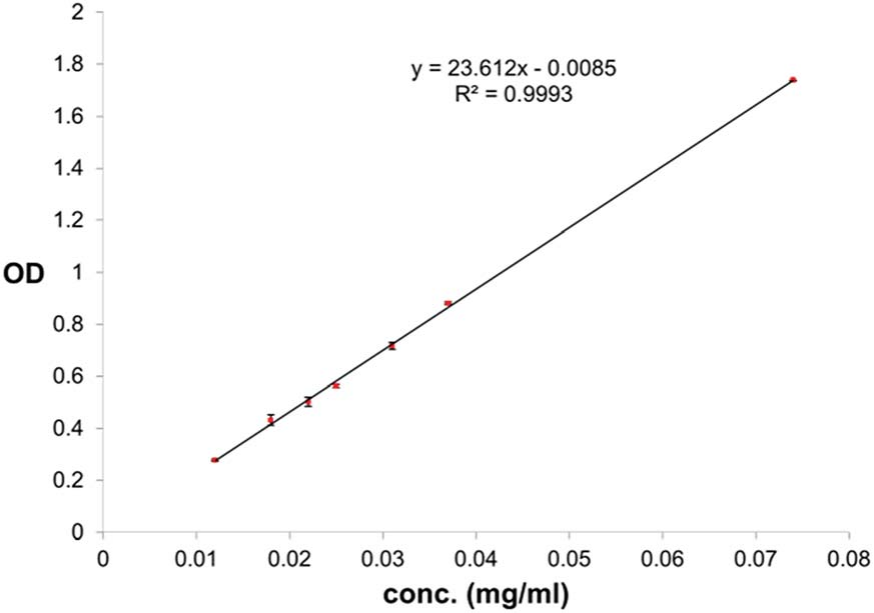
Calibration curve for quantification of carvacrol in NH_4_OH_(aq)_ through UV absorption at 293 nm.

For quantification of carvacrol in NPs through UV absorption, lyophilized NPs were suspended in NH_4_OH_(aq)_ (25–30%) to a concentration of 0.3–1.3 mg NPs/ml NH_4_OH_(aq)_. The suspension was kept in a shaker bath at 60 °C for 24 hours to dissolve the silica and release the carvacrol. The concentration of carvacrol was then determined through use of a calibration curve of carvacrol in NH_4_OH_(aq)_ (25–30%) at 293 nm (Fig. 3). The measurements were done on a Cary 100 Bio UV-visible spectrophotometer of Varian Inc. The conditions were a scan rate of 600.00 nm min^−1^, an average time of 0.100 s, a data interval of 1.000 nm, in double beam mode.

XPS measurements were performed on a Kratos Axis HS spectrometer equipped with an Al Kα X-ray radiation source (photon energy of 1486.6 eV). The pass energy was 80 eV for survey spectra and 40 eV for HR spectra. The source power was 75 or 150 W. The binding energies (BE) of all elements were re-calibrated by setting the CC/CH component of the C 1s peak at 285 eV. For quantitative analysis high-resolution core-level spectra were used and Shirley background correction performed. Spectra analysis and deconvolution was performed with the Vision Software (Kratos). Overlapping signals were analyzed after deconvolution into Gaussian/Lorentzian-shaped components.

## Results and discussion

### Synthesis of carvacrol-(3-(triethoxysilyl)propyl)carbamate (CTESPC)

Carvacrol possesses a phenolic hydroxyl group, enabling silanization through formation of a urethane (carbamate) linkage with a silanated isocyanate (in this case 3-(triethoxysilyl)-propyl isocyanate). An efficient catalyst for urethane formation is tetraoctyltin, a Lewis acid catalyst forming a catalytically active complex with alcohols and isocyanates, both of which are electron donors. The bulky octyl substituents seem to depress complexation of the resulting carbamate with the electron-accepting tin atoms, which would reduce the concentration of active catalyst.^44^ Previously, this tetraoctyltin-catalyzed carbamoylation reaction has been utilized to silanate the biocide triclosan.^45^

We confirmed the formation of CTESPC (silanated carvacrol) through ^1^H-NMR, ^13^C-NMR, Fourier-Transform Infrared (FT-IR), and UV-VIS analyses, and mass spectroscopy.

The ^1^H-NMR spectrum of the product purified through silica column chromatography shows the broad triplet of the carbamate NH-proton at 5.35 ppm and the presence of the methylene and methyl protons of the silane aliphatic groups at 3.84, 3.28, 1.71, 1.24, and 0.69 ppm (TMS as reference) with the correct integrations and multiplicities. The ^13^C-NMR spectrum contains the corresponding carbon peaks of aliphatic carbons at 58.5, 43.6, 23.9, 15.7, and 7.7 ppm (TMS as reference). The DEPT 135 (Distortionless Enhancement by Polarization Transfer) spectrum identifies the peaks at 58.5, 43.6, 23.9, and 7.7 ppm as peaks of methylene carbons, which confirms the covalent attachment of the silane moiety originating from the isocyanate.

**Fig. 4:**
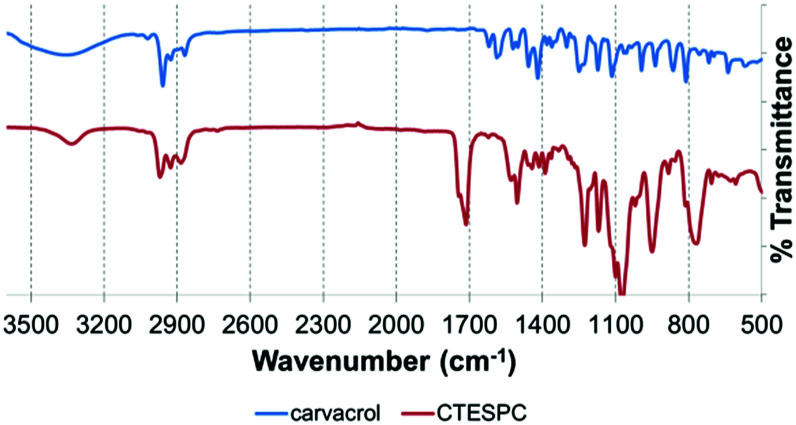
Comparison of FT-IR spectra of free carvacrol (upper curve) and CTESPC (lower curve).

In the FT-IR spectrum of CTESPC (Fig. 4), the absorption of the C=O stretch of the urethane linkage appears at 1714 cm^−1^ (s) and the N–H stretch at 3334 cm^−1^ (w). Free carvacrol shows a broad peak centered around 3300 cm^−1^ (s) of the phenolic OH-stretch with hydrogen-bonding, which does not appear in CTESPC. At 1100 cm^−1^ (m) and 1074 cm^−1^ (s), there is a strong, broad peak with a typical doublet shape which is also seen in the spectrum of TEOS (not shown), confirming silane formation. According to the wavenumbers, these peaks can be assigned to a Si–O stretch of silicon alkoxides (1000–1100 cm^−1^ (s))^46^ or to the C–O stretch of the ethoxide group (1050–1175 cm^−1^).^47^ Some sources assign 1074 cm^−1^ to both groups, while some assign both peaks, 1100 and 1074 cm^−1^, to the C–O stretch.^48^ At 813 and 769 cm^−1^ (m), we see a similarly-shaped, but weaker peak. In the spectrum of TEOS, there is one peak at 785 cm^−1^. It is attributed to the Si–O or the C–O stretch.^48^ The broader shape with the double peak here might be caused by the additional absorption of the Si–C stretch (700–820 cm^−1^ (m))^46^ which is not found in TEOS.

The mass spectrum in electrospray ionization shows a strong peak at *m*/*z* of 420.2, belonging to M^+^ + Na. It confirms the molecular weight, which is calculated as 397.58 amu.

**Fig. 5:**
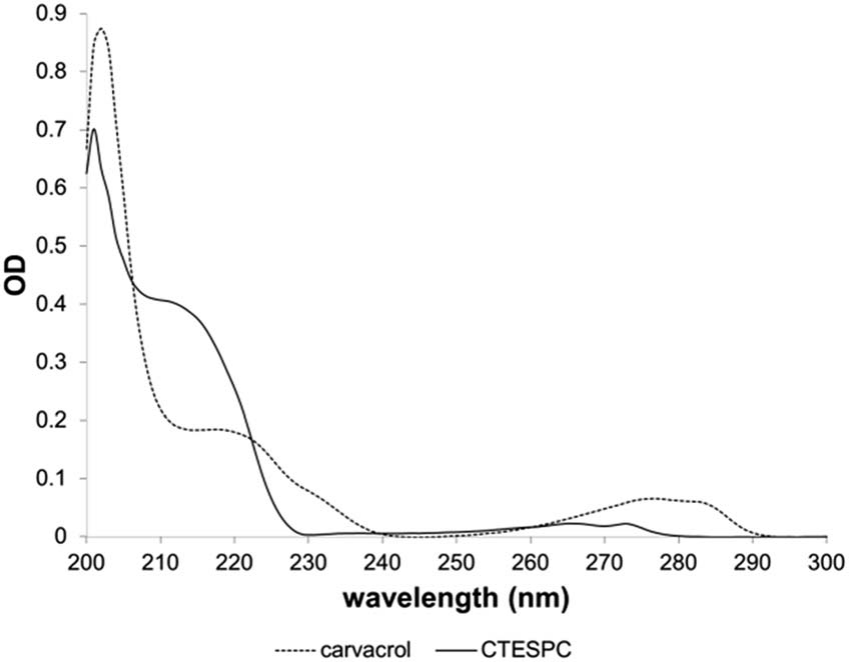
Comparison of UV absorption spectrum of carvacrol and CTESPC.

The UV absorption spectrum (Fig. 5) shows a shift of the plateau at 218 nm (free carvacrol) to 211 nm (CTESPC) and the local absorption maximum at 276 nm to ∼265 nm, which confirms covalent modification of carvacrol.

### Optimization of fabrication of NPs with covalently-bound carvacrol – TEM and DLS diameter and *ζ*-potential

When the NPs are in the presence of pathogens, the bacterial membrane esterases can hydrolyze the C–O single bond of the urethane group of the NPs. Then, carvacrol located on the surface of the particles is released. For that purpose, the NPs need to be in contact with the bacterial cell membrane. This contact increases interaction between enzymes and the NP surface, beyond what is possible by diffusion alone. To maximize contact, the NPs should be stable and not too large because aggregation and large size reduce the NP surface area. Because EO compounds have an MBC (minimum bactericidal concentration) that is by several orders of magnitude higher than the MBC of conventional antibiotic agents, the concentration of CTESPC on the NP surface needs to be high. However, a high concentration of organic content affects the NPs' properties.

**Fig. 6:**
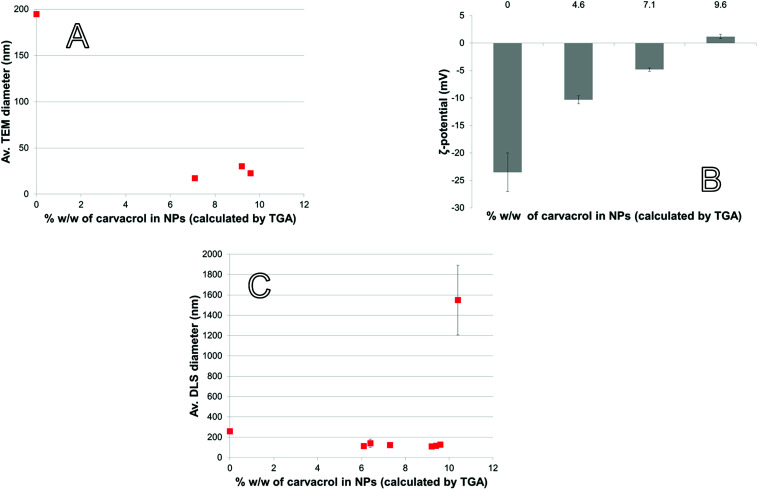
NP properties as a function of carvacrol content: (A) TEM diameter, (B) *ζ*-potential (measured in EtOH), (C) DLS diameter (measured in EtOH).

With large amount of carvacrol in the NPs in % w/w, the Transmission Electron Microscopy (TEM) size decreases to below 50 nm (Fig. 6A). CTESPC, especially the isopropyl group, seems to cause steric hindrance of the reactive sites for condensation that prevents further NP growth and leads to enrichment of the NP surface in CTESPC, which is desirable. This is also evident from the fact that *ζ*-potential decreases with increasing % w/w of carvacrol (Fig. 6B) because the density of charged silanol groups on the surface decreases. The downside of this is that low *ζ*-potential means lower electrostatic repulsion and the NPs are more prone to aggregate. The organic groups on the NP surface form van der Waals, hydrophobic, and π-stacking interactions with organic groups on other NPs. Aggregation manifests itself in large DLS size (Fig. 6C) and large standard deviation at >10% w/w carvacrol. An additional reason for the observed changes may be the decrease in H_2_O : Si and NH_4_OH : Si ratios with increasing CTESPC concentration in the reaction mixture that affect the degree of hydrolysis and condensation. Also, CTESPC possibly undergoes less complete hydrolysis than TEOS.

**Fig. 7:**
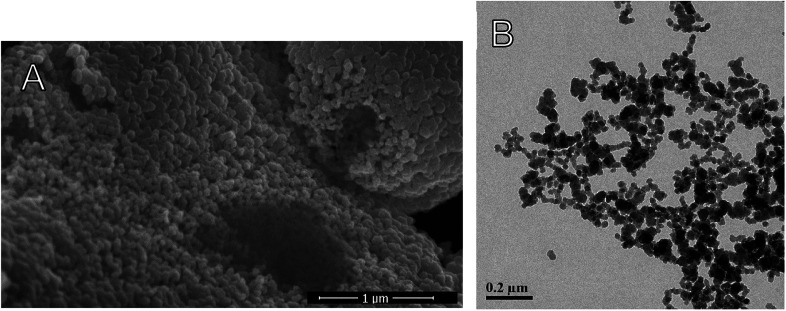
(A) High Resolution Scanning Electron Microscopy (HR-SEM) of dried CTESPC-containing NPs (fabricated at 25% *n*/*n*, ∼10.4% w/w carvacrol) (scale bar 1 μm), (B) TEM of CTESPC-containing NPs (fabricated at 17% *n*/*n*, ∼9.6% w/w carvacrol, av. diameter in TEM 22.9 nm, DLS diameter 127.8 ± 21.0 nm) (scale bar 0.2 μm).

High CTESPC concentration leads to phase separation in the reaction mixture at RT due to super-saturation of the solution. We, therefore, performed co-condensation at 60 °C.

**Fig. 8:**
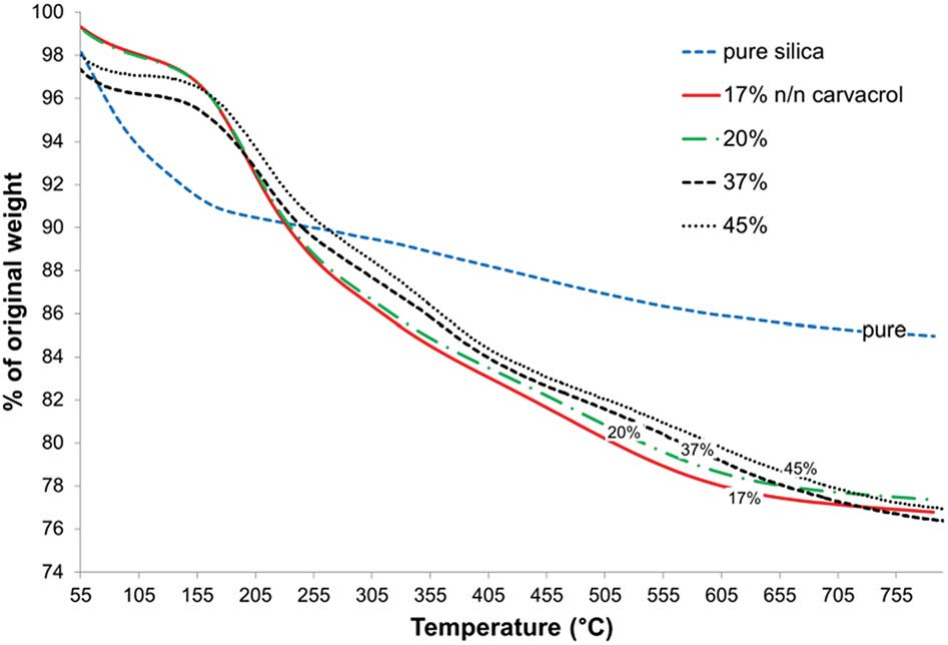
Comparison of TGA curves of pure silica and of several samples of carvacrol-containing NPs fabricated with different concentrations of CTESPC in the reaction mixture.

CTESPC concentration ≥ 35% *n*/*n* (see Experimental) leads to low yield of NPs expressed as mg NPs/ml, and at CTESPC concentrations ≤ 35% *n*/*n* the maximum % w/w of carvacrol in the NPs is on average reached from 17–20% *n*/*n* on. Further increase in CTESPC concentration in the reaction mixture does not lead to significant increase in the amount of CTESPC incorporated in the NPs. On the contrary, it can lead to a slight decrease in CTESPC content. This state of saturation is seen in Fig. 8, which shows the TG curves of several samples of hybrid-silica NPs fabricated with high concentration of CTESPC in the reaction mixture. Therefore, 17–20% *n*/*n* CTESPC was chosen as optimal concentration.

### Quantification of carvacrol in NPs and on NP surface – TGA/DSC and XPS

TGA can be used to quantify organic content in an inorganic matrix that does not undergo pyrolysis or combustion in the range of temperatures employed. In the case of hybrid-silica materials, quantification is complicated by the presence of organic content from incomplete hydrolysis in addition to the organic moiety that we intend to quantify. In addition, decrease in weight from adsorbed water and solvent needs to be distinguished from weight loss due to the organic content. Therefore, a thorough analysis of the corresponding weight loss curves is required to correctly estimate the weight loss attributed to the organo-silicate component. Fig. 8 confirms the hydrophobic organic nature of the NP surface because the initial weight loss due to adsorbed and entrapped water and ethanol is much lower for the carvacrol-containing silica than for pure silica. This is expressed in the flat slope at the lower temperatures.

**Fig. 9:**
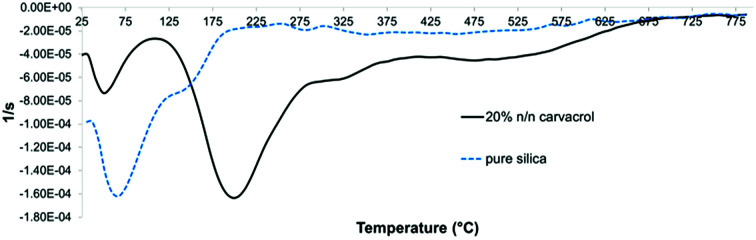
DTG curves (first derivatives of the TG curves) of pure silica and CTESPC-containing hybrid silica fabricated at 20% *n*/*n* CTESPC (the same sample as in Fig. 8).

The first derivative of the TGA curve (called DTG), which expresses the rate of the weight change, was utilized for identification of distinct weight loss events and determining the onset and end of each weight loss event. In addition, integration of the peak area quantifies the weight loss. Fig. 9 shows the DTG curves of non-hybrid (pure) silica NPs and hybrid-silica NPs with high carvacrol content. We can distinguish two major weight loss events. In pure silica NPs, the first event (loss of water and solvent mostly) ends at about 195 °C. It is obvious from the curve that this event constitutes most of the weight loss of silica NPs. For hybrid-silica with high organic content, the first weight loss step ends at 110 °C, and the major weight loss is in this case the second event (the decomposition of the organo-silicate). For quantification of carvacrol in the NPs, we did not differentiate between the weight loss events after the initial one since they all pertain to the decomposition of organic groups. Therefore, we called the range of initial weight loss “Step 1” and the range of the remaining weight loss “Step 2”. We subtracted from the weight loss of Step 2 the average weight loss of pure silica NPs in Step 2, which is probably mostly due to ethoxy groups that did not undergo hydrolysis. This is under the assumption that the average degree of hydrolysis of silica formed from TEOS and hybrid silica formed from TEOS and CTESPC is similar.

**Fig. 10:**
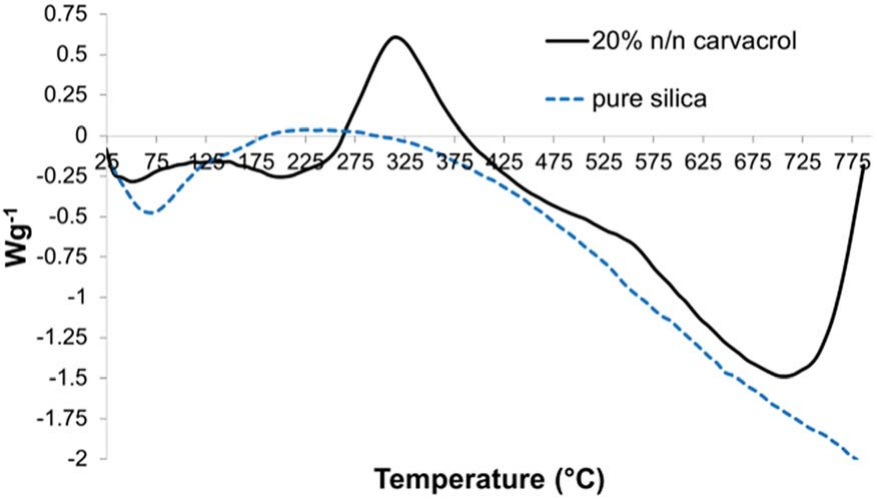
DSC curves of the same samples as in Fig. 9.

The Differential Scanning Calorimetry (DSC) curves served to identify endothermic and exothermic events and their temperature ranges. The DSC curve of hybrid-silica with carvacrol (Fig. 10) shows an exotherm with a maximum at around 325 °C that is absent in the curve of silica NPs. We assume it corresponds to chemical reactions of the organo-silicate groups while decomposing.

**Fig. 11:**
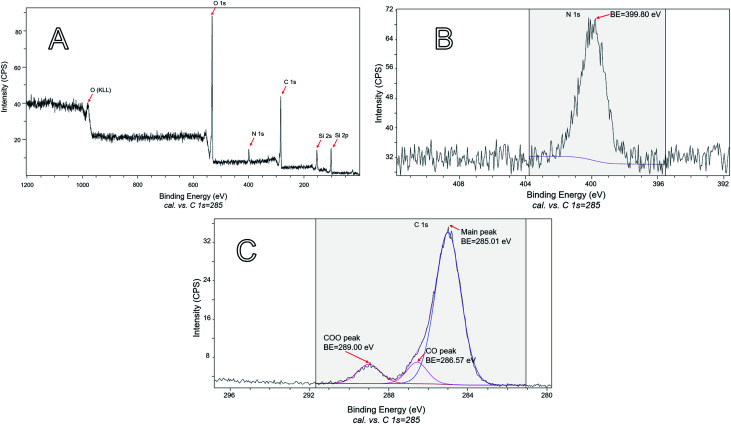
XPS: (A) survey scan of the sample shown in Fig. 7A. (B) The part of the high-resolution scan that shows the N 1s emission of the sample shown in Fig. 7A. (C) The part of the high-resolution scan that shows the C 1s emissions of the sample shown in Fig. 7A.

Because only carvacrol that is on the NP surface contributes to the antibacterial effect, it is especially important to characterize the composition of the surface (although it is expected that the composition of the bulk of the NPs reflects the surface composition, too). Therefore, XPS analysis was performed on samples of hybrid silica containing a large percentage of CTESPC. The presence of CTESPC on the surface of the particles was confirmed through the peaks of nitrogen N 1s (BE of 399.80 eV) (Fig. 11B) and the 3 peaks of carbon C 1s (Fig. 11C). The small C 1s peak at highest binding energy (BE: 289.00 eV) is caused by electron emission from the highly oxidized carbonyl carbon of the urethane linkage. The main peak of C 1s is asymmetric because it consists of 2 overlapping peaks: the peak of aliphatic and aromatic carbons (BE: 285.01 eV) and at higher binding energy the peak of the phenolic carbon atom (BE: 286.57 eV). Peak-fitting was done to deconvolute that peak. These values are in the range of values in the literature: 285 eV for C 1s of carbon atoms with C–C or C–H bonds, 286.2–288.0 eV for C 1s of ethers, and 288.0–289.2 eV for C 1s peaks of COO.^49^

The ratios of surface atoms in the carvacrol-containing NPs are summarized in Tables 1 and 2.

**Table 1:** Quantitative XPS results of all peaks

	O 1s	N 1s	Si 2p	C 1s
% atomic concentration	25.61	3.43	16.93	54.03
Atom ratio	7.5	1	5	16

**Table 2:** Quantitative XPS results of C 1s peaks

	C 1s main	C 1s COO	C 1s CO
% atomic concentration	81.55	9.26	9.19
Atom ratio	9	1	1

To show the atom ratio, the atomic concentration expressed as percentage was divided by the lowest percentage, which was defined as representing one atom and rounded. Table 2 shows that the ratio of the oxidized carbon atoms (COO : CO = 1 : 1) is the correct ratio found in CTESPC.

For each molecule of CTESPC represented by 1 nitrogen, 14 carbon, 1 silicon, and 3.5 oxygen atoms (assuming complete hydrolysis and condensation), there were 4 additional oxygen, 2 carbon, and 4 silicon atoms (see Table 1) which originate approximately from 4 TEOS molecules. That means that for every 4 TEOS molecules bound 1 CTESPC molecule is incorporated. This constitutes a high percentage of CTESPC at the top 5–10 nm. Testing several carvacrol NP samples proved that this result is consistent.

### Preliminary biological results

**Fig. 12:**
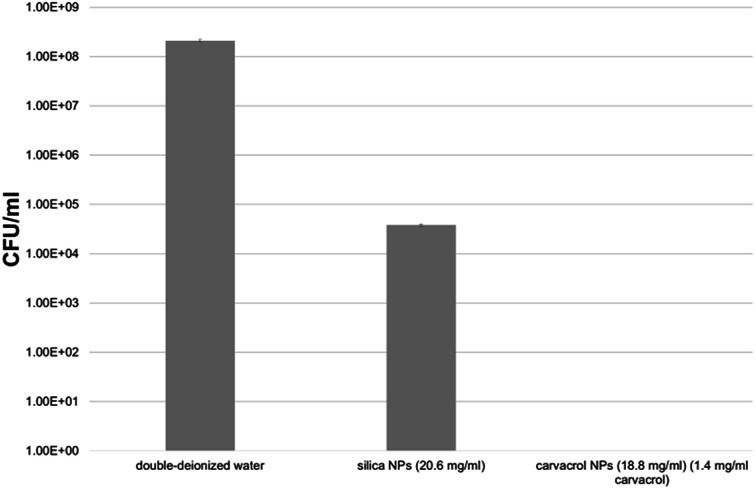
Results of incubation of CTESPC-containing NPs with *E. coli* for 24 hours.

We tested the antibacterial activity of the carvacrol-containing hybrid NPs after extensive washing and centrifugation cycles (9–10 cycles, see Experimental) to ensure that no trace of unreacted carvacrol-(3-(triethoxysilyl)propyl)carbamate (CTESPC) was present. *E. coli* bacteria were incubated for 24 hours with several NP samples fabricated at ∼17% *n*/*n* carvacrol whose carvacrol content had been quantified by TGA and UV absorption. It was determined that the concentration required to kill all the bacteria was 1.4 mg ml^−1^ carvacrol contained in NPs which required a hybrid silica NP concentration in the range of 14–19 mg ml^−1^ NPs. This seems to be higher than the MBC (minimal bactericidal concentration) of carvacrol in water (determined by us as 0.35 mg ml^−1^), which is comparable to 25% of the quantity of carvacrol contained in the copolymeric NPs. However, the concentration of carvacrol in the NP solution that is actually active is expected to be lower since all carvacrol is covalently bound and needs to be released by hydrolytic cleavage to become active. This needs close contact between the bacterial cell wall and each NP. Moreover, the copolymeric NPs contain also carvacrol that is not on the surface or is not sterically accessible and therefore is not available for esterases. Pure silica NPs (that is, without carvacrol) showed a certain antibacterial effect, too, but the presence of carvacrol enhances the effect by log 4.5 compared to pure silica, which shows even at slightly higher concentration a much smaller effect, as can be seen in Fig. 12. The advantage of use of the NPs as opposed to pure carvacrol (no smell and no vapor pressure, low toxicity, sustained release on demand, improved long-term stability and storage properties, better dispersion due to higher hydrophilicity) are discussed in Conclusions.

## Conclusions

We successfully synthesized CTESPC, a silane reagent incorporating the phenolic antibacterial compound carvacrol as organic moiety. We then used CTESPC as precursor for the fabrication of NPs that contain carvacrol covalently-bound with a carbamate bond that is enzymatically hydrolyzable. As a result, the NPs can function as slow-release antibiotics on demand with very little carvacrol released at a time, therefore presumably active for extended periods. Because of the low toxicity of carvacrol and its low concentration due to the slow-release mechanism further reducing the toxicity, the hybrid-silica NPs can be considered a “soft antibiotic”. The silica matrix protects the biocide from the degrading effects of UV light, moisture, and exposure to air and heat, which should improve the stability during long-term storage. At the same time the silica matrix also imparts some hydrophilicity to the NPs for their optimal dispersion in water-based media. An important advantage in applications is that the NPs do not contain any unbound carvacrol. Therefore, they have no volatile component and have no smell. This is opposed to the very distinct and strong smell of pure carvacrol in water, which would be undesirable in consumer products and would preclude many applications.

Carvacrol has GRAS status, and the carvacrol used was even FG (Food-Grade). Amorphous silica is also considered safe and nontoxic and is already used in a wide range of consumer applications, including toothpastes, cosmetics, and even food.^40,41^ Therefore, the antibiotic hybrid-silica NPs that we developed can be used in applications where silica is already present and where an antibacterial effect would be advantageous. Other potential applications include preservatives, dental antibacterial materials, and use in water purification filters.

The one-step carbamoylation reaction that produces CTESPC gives relatively good yields and is easily scaled up. Co-condensation, too, is a simple fabrication method that can easily be adapted to large-scale production and needs no complicated equipment. The antibacterial NPs are expected to be very stable as dry powder or in solution in ethanol or water at neutral pH (which does not encourage spontaneous hydrolysis).

The carbamoylation reaction we employed to synthesize the organo-silane incorporating the antibacterial compound can be readily extended to other phenolic antibacterial compounds, especially other natural terpenoids.

## Conflicts of interest

There are no conflicts to declare.

## Supplementary Material

## References

[cit1] World Health Organization , Antimicrobial Resistance: Global Report on Surveillance, WHO Press, Geneva, 2014

[cit2] Bakkali F., Averbeck S., Averbeck D., Idaomar M. (2008). Food Chem. Toxicol..

[cit3] Food and Drug Administration , CFR – Code of Federal Regulations Title 21, http://www.accessdata.fda.gov/scripts/cdrh/cfdocs/cfcfr/CFRSearch.cfm?FR=172.515, accessed, July, 2015

[cit4] Kalemba D., Kunicka A. (2003). Curr. Med. Chem..

[cit5] U.S. Food & Drug Administration , FDA issues final rule on safety and effectiveness of antibacterial soaps, https://www.fda.gov/newsevents/newsroom/pressannouncements/ucm517478.htm, accessed, November, 2017

[cit6] Buth J. M., Steen P. O., Sueper C., Blumentritt D., Vikesland P. J., Arnold W. A., Mc Neill K. (2010). Environ. Sci. Technol..

[cit7] Dhillon G. S., Kaur S., Pulicharla R., Brar S. K., Cledon M., Verm M., Surampalli R. Y. (2015). Int. J. Environ. Res. Public Health.

[cit8] Hammer K. A. and Carson C. F., in Lipids and Essential Oils as Antimicrobial Agents, ed. H. Thormar, John Wiley & Sons, Ltd, Chichester, UK, 2011, ch. 11

[cit9] Langeveld W. T., Veldhuizen E. J. A., Burt S. A. (2014). Crit. Rev. Microbiol..

[cit10] Yap P. S. X., Yiap B. C., Ping H. C., Lim S. H. E. (2014). Open Microbiol. J..

[cit11] Palaniappan K., Holley R. A. (2010). Int. J. Food Microbiol..

[cit12] Burt S. (2004). Int. J. Food Microbiol..

[cit13] Ultee A., Bennik M. H. J., Moezelaar R. (2002). Appl. Environ. Microbiol..

[cit14] Nostro A., Roccaro A. S., Bisignano G. (2007). J. Med. Microbiol..

[cit15] Dalleau S., Cateau E., Berges T. (2008). Int. J. Antimicrob. Agents.

[cit16] Hirasa K. and Takemasa M., Spice Science and Technology, Dekker, Inc., New York, 1998

[cit17] Piaru S. P., Mahmud R., Abdul Majid A. M., Mahmoud Nassar Z. D. (2012). Asian Pac. J. Trop. Med..

[cit18] Chen W., Gao M., Wu J., Wang A., Shi R. (2011). J. Ethnopharmacol..

[cit19] Yoo C. B., Han K. T., Cho K. S., Ha J., Park H. J., Nam J. H., Kil U. H., Lee K. T. (2005). Cancer Lett..

[cit20] Carson C. F. and Hammer K. A., in Lipids and Essential Oils as Antimicrobial Agents, ed. H. Thormar, John Wiley & Sons, Ltd, Chichester, UK, 2011, ch. 9

[cit21] Bishop C. D. (1995). J. Essent. Oil Res..

[cit22] Azzouz M. A., Bullerman L. B. (1982). J. Food Prot..

[cit23] Akgul A., Kivanc M., Sert S. (1991). Sci. Aliments.

[cit24] Jayashree T., Subramanyam C. (1999). Lett. Appl. Microbiol..

[cit25] Mari M., Bertolini P., Pratella, G. C. (2003). J. Appl. Microbiol..

[cit26] Pandey R., Kalra A., Tandon S., Mehrotra N., Singh H. N., Kumar S. (2000). J. Phytopathol..

[cit27] Pessoa L. M., Morais S. M., Bevilaqua C. M. L., Luciano J. H. S. (2002). Vet. Parasitol..

[cit28] Konstantopoulou L., Vassilopoulo L., Mavragani-Tsipidou P., Scouras Z. G. (1992). Experientia.

[cit29] Karpouhtsis I., Pardali E., Feggou E., Kokkini S., Scouras Z. G., Mavragani-Tsipidou P. (1998). J. Agric. Food Chem..

[cit30] Wattanasatcha A., Rengpipat S., Wanichwecharungruang S. (2012). Int. J. Pharm..

[cit31] Shah B., Davidson P. M., Zhong Q. (2013). Int. J. Food Microbiol..

[cit32] Pan K., Chen H., Davidson P. M., Zhong Q. (2014). J. Agric. Food Chem..

[cit33] Sokolik C. G., Ben-Shabat-Binyamini R., Gedanken A., Lellouche J. P. (2018). Ultrason. Sonochem..

[cit34] Ashraf M. A., Khan A. M., Ahmad M., Sarfraz M. (2015). Front. Chem..

[cit35] Vega O., Araya J. J., Chavarria M., Castellon E. (2016). J. Sol-Gel Sci. Technol..

[cit36] Paseta L., Simon-Gaudo E., Gracia-Gorria F., Coronas J. (2016). Chem. Eng. J..

[cit37] Chen F., Shi Z., Neoh K. G., Kang E. T. (2009). Biotechnol. Bioeng..

[cit38] Ruiz-Rico M., Pérez-Esteve E., Bernardos A., Sancenón F., Martínez-Máñez R., Marcos M., Barat J. M. (2017). Food Chem..

[cit39] Colilla M. and Vallet-Regi M., in Comprehensive Biomaterials, ed. P. Ducheyne, Elsevier, Amsterdam, 2011, vol. 1, ch. 4.429

[cit40] Contada C., Mejia J., Gracia O. L., Piret J. P., Dumortier E., Toussaint O., Lucas S. (2016). Anal. Bioanal. Chem..

[cit41] Merget R., Bauer T., Kuepper H. U., Philippou S., Bauer H. D., Breitstadt R., Bruening T. (2002). Arch. Toxicol..

[cit42] Raileanu M., Todan L., Voicescu M., Ciuculescu C., Maganu M. (2013). Mater. Sci. Eng., C.

[cit43] Food and Drug Administration , Select Committee on GRAS Substances (SCOGS) Opinion: Clove Bud Oil, http://www.fda.gov/Food/IngredientsPackagingLabeling/GRAS/SCOGS/ucm261255.htm, accessed, July, 2015

[cit44] Thiele L. and Becker R., in Advances in Urethane Science and Technology, ed. K. C. Frisch and D. Klempner, Technomic Publishing, Lancaster, Pennsylvania, 1993, vol. 12, p. 59

[cit45] Makarovsky I., Boguslavsky Y., Alesker M., Lellouche J., Banin E., Lellouche J. P. (2011). Adv. Funct. Mater..

[cit46] Robinson J. W. , Skelly Frame E. M. and Frame G. M., Undergraduate Instrumental Analysis, CRC Press, New York, NY, 2005

[cit47] Yurkanis Bruice P. , Organic Chemistry, Pearson Education, Inc., Upper Saddle River, NJ, 2007

[cit48] Tejedor-Tejedor M. I., Paredes L., Anderson M. A. (1998). Chem. Mater..

[cit49] Database, XPS (X-ray Photoelectron Spectroscopy) , XPS Spectra – Chemical Shift|Binding Energy, http://techdb.podzone.net/xpsstate-e/, accessed, October, 2015

